# Clinical Recognition, Safe Triage, and Pharmacovigilance Reporting Readiness for Suspected Serious Adverse Drug Reactions in Community Pharmacies: A Covert Simulated-Patient Study in Central Jordan

**DOI:** 10.3390/jcm15218652

**Published:** 2026-11-06

**Authors:** Zekrayat J. H. Merdas, Mohammad Abu Assab, Anas Abed, Wala’a Al. Safadi, Zain Z. Zakaria, Zainab Zakaraya, Sireen Abdul Rahim Shilbayeh

**Affiliations:** 1 Pharmacy Department, College of Pharmacy, Amman Arab University, Amman 11953, Jordan; th.merdas@aau.edu.jo; 2 Clinical Pharmacy Department, Faculty of Pharmacy, Zarqa University, Zarqa 13132, Jordan; mabuassab@zu.edu.jo; 3 Department of Biopharmaceutics and Clinical Pharmacy, Faculty of Pharmacy, Al-Ahliyya Amman University, Amman 19111, Jordan; z.zakaraya@ammanu.edu.jo; 4 Department of Biopharmaceutics and Clinical Pharmacy, Faculty of Pharmacy, The University of Jordan, Amman 11942, Jordan; wla9250269@ju.edu.jo; 5 Clinical Affairs Department, Qatar University, Doha 2713, Qatar; zain.zakaria@qu.edu.qa; 6 Department of Pharmacy Practice, College of Pharmacy, Princess Nourah bint Abdulrahman University, P.O. Box 84428, Riyadh 11671, Saudi Arabia; ssabdulrahim@pnu.edu.sa

**Keywords:** adverse drug reaction, medication safety, clinical recognition, safe triage, community pharmacy, pharmacovigilance, simulated patient, Jordan

## Abstract

**Background/Objectives:** Serious adverse drug reactions (ADRs) may first present with nonspecific symptoms outside hospital settings, where delayed recognition or escalation can postpone definitive care. Community pharmacists are often a first point of contact, but behavior-based evidence on their clinical recognition and triage of serious ADRs is limited. We evaluated clinical assessment, recognition, safe triage, and subsequent pharmacovigilance reporting readiness across three high-risk presentations. **Methods:** A cross-sectional covert simulated-patient study was conducted in 384 urban community pharmacies in Amman, Zarqa, and Al-Balqa, Jordan. Pharmacies were randomly assigned one of three standardized scenarios: semaglutide-associated acute pancreatitis, empagliflozin-associated euglycemic diabetic ketoacidosis, or apixaban-associated gastrointestinal bleeding. The primary outcome was a guideline-informed safe-triage response requiring urgent ED/EMS referral, explicit communication of urgency, and no management likely to delay care; pharmacovigilance reporting behavior and readiness were assessed separately after completion of the clinical consultation. **Results:** Safe triage occurred in 41.7% of encounters (design-based 95% CI, 37.0–46.3), with higher performance for apixaban (60.9%) than semaglutide (42.2%) and empagliflozin (21.9%; overall *p* < 0.001). A medication-related cause was recognized in 72.9%, whereas the specific syndrome or harm was named in 39.6%; unsafe management likely to delay assessment occurred in 26.3%. Only 8.3% spontaneously raised reporting. In adjusted analysis, safe triage was less prevalent in the empagliflozin scenario (aPR 0.51, 95% CI 0.35–0.74) and more prevalent in the apixaban scenario (aPR 1.54, 95% CI 1.21–1.96) than in the semaglutide reference scenario. High workload was associated with lower safe-triage prevalence, whereas a visible consultation area and recent pharmacovigilance training were associated with higher prevalence. **Conclusions:** In urban community pharmacies in central Jordan, important gaps were observed in recognition, explicit escalation, and safe triage, particularly for the euglycemic ketoacidosis presentation. Spontaneous pharmacovigilance engagement was also limited. Interventions should prioritize drug-specific red-flag recognition, unambiguous escalation language, workflow support for focused assessment, and low-burden reporting after immediate patient safety has been addressed. These findings should not be generalized to rural pharmacies or other regions of Jordan without further evaluation.

## 1. Introduction

Pharmacovigilance encompasses the detection, assessment, understanding, and prevention of adverse effects and other medicine-related problems [1]. Adverse drug reactions (ADRs) are an important source of preventable clinical harm because they can precipitate emergency visits, hospital admission, prolonged hospitalization, and death [2]. The clinical challenge is not limited to identifying a medicine–event relationship after the fact: serious ADRs may initially resemble common symptoms, and delayed recognition or escalation can postpone definitive assessment. Spontaneous reporting remains susceptible to substantial under-reporting [3], but effective medication safety begins with clinical recognition and appropriate immediate management at the point where medicine-related harm first becomes visible.

Community pharmacists are positioned at this early clinical interface. They are accessible without appointment and routinely encounter patients seeking advice about new symptoms, recently started or dose-adjusted medicines, self-medication, and treatment-related concerns. Their medication-safety role is therefore sequential: identify a potentially serious medicine-related presentation, communicate the need for timely escalation, avoid advice that could delay care, and, once the immediate risk has been addressed, contribute the suspected event to pharmacovigilance. Educational interventions can improve ADR reporting, although gains may be difficult to sustain without practical procedures, reminders, feedback, and organizational support [4]. Across Arab and Middle Eastern countries, pharmacovigilance capacity has expanded, but professional participation and reporting infrastructure remain variable [5,6]. Recent community-pharmacy studies in Saudi Arabia and Yemen similarly describe gaps in pharmacovigilance knowledge or practice [7,8].

Jordan has an established national pharmacovigilance function within the Jordan Food and Drug Administration (JFDA), including an electronic reporting route that accepts suspected ADR reports from pharmacists [9]. National data demonstrate that clinically important ADRs are being captured: analyses of the JFDA database documented increasing reporting over 2010–2014 [10], while a later analysis of 2015–2021 reports found that more than one quarter were classified as serious [11]. A tertiary teaching-hospital registry subsequently demonstrated substantially greater reporting activity within an active institutional system [12]. These data characterize reported events, but they do not show how suspected serious ADRs are recognized and managed when patients first seek help in community practice.

Evidence from Jordanian community pharmacists has mainly relied on self-reported knowledge, attitudes, and practice. Earlier work identified important gaps in pharmacovigilance knowledge and ADR-reporting practice [13], and more recent surveys have continued to show incomplete understanding of reportability and low reporting engagement [14,15]. Qualitative work has identified insufficient training, procedural complexity, lack of feedback, and limited professional recognition as barriers [16]. These studies clarify perceived determinants, but they cannot establish whether a pharmacist confronted with a clinically credible presentation recognizes the potential ADR, identifies the seriousness of the syndrome, communicates urgency, or avoids management that might delay care.

The covert simulated-patient method can directly evaluate these behaviors under routine service conditions [17]. Reviews support its use in community-pharmacy research while emphasizing scenario validation, simulated-patient standardization, detection assessment, ethical safeguards, and transparent reporting [18,19]. Jordanian simulated-patient studies have demonstrated feasibility across medicine-information requests, insomnia, pregnancy-related nausea and vomiting, and topical corticosteroid stewardship [20,21,22,23]. However, these studies have largely focused on counseling, product supply, or condition management rather than the clinical recognition and escalation of potentially serious medicine-related harm.

This study therefore evaluated community pharmacists’ real-world responses to three suspected serious ADR presentations involving widely used metabolic and cardiovascular therapies: semaglutide-associated acute pancreatitis, empagliflozin-associated euglycemic diabetic ketoacidosis, and apixaban-associated gastrointestinal bleeding. The primary objective was to estimate the proportion of encounters receiving guideline-informed safe triage. Secondary objectives were to compare clinical assessment, medicine-related and syndrome-specific recognition, medication advice, and unsafe management across scenarios; to distinguish spontaneous pharmacovigilance behavior from reporting readiness after a standardized probe; and to identify pharmacy-, pharmacist-, and visit-level factors associated with safe triage. We hypothesized that the overt bleeding presentation would be managed more reliably than the two metabolic presentations and that recent pharmacovigilance training and supportive workflow characteristics would be associated with better clinical performance.

## 2. Materials and Methods

### 2.1. Study Design and Reporting

We conducted a cross-sectional observational study using covert simulated-patient (SP) visits to urban community pharmacies in Amman, Zarqa, and Al-Balqa, three governorates in central Jordan, between 12 January and 30 April 2026. Each pharmacy received one unannounced visit and one of three scenarios. The unit of analysis was the pharmacy encounter with the licensed pharmacist who provided the consultation. The study was deliberately restricted to urban pharmacy settings; rural and semi-rural areas were outside the sampling scope. The protocol and manuscript were developed with the Strengthening the Reporting of Observational Studies in Epidemiology (STROBE) statement and the refined Checklist for Reporting Research Using a Simulated Patient Methodology in Health (CRiSPHe) [24,25].

### 2.2. Setting, Sampling Frame, and Eligibility

The source registry was a Jordan Pharmacists Association (JPA) extract obtained and frozen on 5 January 2026, containing 5048 licensed community-pharmacy outlets nationwide [26]. The analytic sampling frame was restricted to 3580 eligible urban outlets in Amman (n = 2350), Zarqa (n = 780), and Al-Balqa (n = 450). Urban status followed the Jordan Department of Statistics definition stated in the official urban/rural population table—localities with at least 5000 residents, as defined in the 2015 census—and registry addresses were mapped to the corresponding locality classification before randomization [27]. Hospital pharmacies, mail-order-only services, wholesalers, pharmacies listed as permanently closed, and outlets without a licensed pharmacist in a patient-facing role were ineligible. Pharmacies were stratified by governorate. Proportionate allocation yielded 252 visits (65.6%) in Amman, 84 (21.9%) in Zarqa, and 48 (12.5%) in Al-Balqa. Within each governorate, outlets were placed in a computer-generated random order. The research coordinator released pharmacies sequentially until the prespecified governorate and scenario quotas were filled, with replacements drawn from the next randomly ordered outlet when a selected pharmacy was closed, lacked an available pharmacist, declined post-encounter data retention, or had a protocol deviation. Chain-organization identifiers were retained as de-identified codes solely for sensitivity analysis of within-organization correlation.

A pharmacy was eligible when an SP could speak directly with a licensed pharmacist. When several pharmacists were present, the SP approached the pharmacist who first offered assistance; staff identity and licensure status were confirmed only during debriefing. A selected outlet received up to two attempts at different times. No pharmacy or pharmacist was visited more than once, and pilot pharmacies were excluded from the sampling frame used for the main study.

### 2.3. Sample Size and Scenario Allocation

The prevalence of a safe response was unknown. Using a conservative expected proportion of 50%, a two-sided 95% confidence level, and approximately ±5 percentage-point precision yields a requirement of about 384–385 encounters before finite-population correction. We fixed the target at 384 to permit equal allocation to three scenarios (128 each) while retaining essentially the same precision. The approach list was increased by 43 pharmacies (11.2% relative to the 384-encounter target) to 427 pharmacies. Within each governorate, pharmacies were assigned to scenario quotas using randomly permuted blocks of six. Because each SP was trained for and assigned to one scenario, SPs necessarily knew the scenario they would portray; however, pharmacy identities and daily route assignments were concealed from SPs until morning route release. The study was designed primarily to estimate overall performance within the defined three-governorate urban sampling domain; scenario comparisons and multivariable analyses were interpreted using effect estimates and confidence intervals.

### 2.4. Development of the Clinical Scenarios

Scenario construction followed four principles: clinical seriousness, plausible presentation to a community pharmacy, a recognized medicine–event association, and variation in diagnostic conspicuity. Scripts were written in Jordanian Arabic with standardized English data definitions. Each script contained information to be volunteered initially, information to be provided only if asked, an affect and urgency profile, a fixed closing probe about reporting, and neutral exit rules. The patient carried a photograph of the medicine package and a written medication list so that the suspected product, dose, and treatment timing were available if requested. The complete standardized scripts and SP rules are provided in Supplementary Material S1.

- Scenario A—GLP-1 RA and possible acute pancreatitis: a woman using once-weekly semaglutide reported severe persistent epigastric pain radiating to the back with nausea after a recent dose escalation. She requested an analgesic or antiemetic and initially preferred not to attend hospital. The expected response was recognition of pancreatitis as a possible serious medicine-related event, immediate emergency-department referral, avoidance of symptomatic management that could delay assessment, and advice not to take the next dose until urgently reviewed.
- Scenario B—SGLT2 inhibitor and possible euglycemic diabetic ketoacidosis: a man taking empagliflozin described reduced food and fluid intake during an intercurrent illness, nausea, abdominal pain, marked fatigue, and deep rapid breathing. He stated that a home glucose reading was 165 mg/dL and requested an antacid or oral rehydration product. The expected response was urgent referral despite the absence of marked hyperglycemia, consideration of ketoacidosis, and temporary withholding of the SGLT2 inhibitor pending emergency assessment.
- Scenario C—apixaban and possible gastrointestinal bleeding: a man taking apixaban 5 mg twice daily for atrial fibrillation reported black stools, dizziness, and weakness after using ibuprofen for back pain. He requested a stomach medicine. The expected response was recognition of clinically significant bleeding, immediate emergency referral, and clear advice to avoid further nonsteroidal anti-inflammatory drug use. Because unsupervised interruption of anticoagulation carries thromboembolic risk, advice to stop apixaban was recorded but was not required for the primary outcome.

Clinical anchors were derived from regulatory and professional guidance that was available before fieldwork and from the JFDA pharmacovigilance reporting interface. The JFDA form accepted reports from pharmacists and requested information on suspected medicines, suspected reactions, seriousness, outcomes, and reporter details [9], while the JFDA Individual Case Safety Report guidance specifies that suspected ADRs can be reported by healthcare professionals and defines the minimum information required for a valid report [28]. The study therefore treated a clinically serious suspected medicine-related event as reportable without requiring proof of causality or a novel/unlabelled reaction; the expected behavior was suspicion, harm-minimizing triage, and willingness to initiate a valid report. The European Medicines Agency warns that SGLT2 inhibitor–associated ketoacidosis may occur without very high glucose and recommends stopping the medicine when ketoacidosis is suspected [29]. Semaglutide product information available before fieldwork identified acute pancreatitis as a recognized risk and advised discontinuation when pancreatitis is suspected [30]. Major bleeding on oral anticoagulation requires rapid evaluation and source control, and concomitant nonsteroidal anti-inflammatory medicines increase concern [31]. Scenarios were not designed to test definitive diagnosis.

### 2.5. Content Validation and Pilot Testing

A seven-member panel comprising two clinical pharmacists, a pharmacovigilance specialist, an emergency physician, an endocrinologist, a cardiologist, and a senior community pharmacist independently assessed clinical plausibility, Jordanian linguistic authenticity, essential assessment items, response scoring, and potential ambiguity. Item-level content-validity indices ranged from 0.86 to 1.00, and the average scale-level content-validity index was 0.94, exceeding conventional thresholds for expert agreement [32]. The panel specifically removed any scoring rule that required a definitive causal attribution and clarified that urgent referral and ADR reporting are complementary rather than mutually exclusive actions.

Eighteen pilot visits, six per scenario, were performed between 1 and 10 December 2025 in pharmacies outside the main sample. Debriefing confirmed that symptoms, medication availability, requests, and conversation length were credible. Pilot findings led to three refinements: the SGLT2 scenario included a written glucose value to prevent inconsistent recall; the apixaban scenario specified the last ibuprofen dose; and the reporting probe was shortened to avoid leading the pharmacist toward a particular channel. Pilot observations were not included in the analysis.

### 2.6. Simulated-Patient Recruitment, Training, and Standardization

Six research assistants unknown to the sampled pharmacy teams served as simulated patients (SPs), with two SPs assigned to each scenario. All completed an eight-hour workshop led by a clinical pharmacist and an SP-methodology researcher. Training covered the clinical rationale, exact opening statements, standardized answers, permitted nonverbal behavior, management of unanticipated questions, use of neutral prompts, timing, detection, debriefing, consent, safety, and immediate checklist completion. Rehearsals continued until each SP delivered all mandatory information within the permitted sequence and did not volunteer hidden information prematurely.

For standardization testing, SPs completed 36 video-recorded mock encounters with trained assessors rotating as pharmacists. Two independent raters scored the recordings. Item-level agreement was 88.9–97.2%, item-level Cohen κ values ranged from 0.80 to 0.95, and κ for the primary safe-triage classification was 0.88, indicating strong agreement [33]. Discrepancies were reviewed using the operational manual before fieldwork. Each SP completed 64 main-study encounters, distributed across the three governorates and across visit periods within the SP’s assigned scenario. Because the two SPs assigned to a given scenario were structurally nested within that scenario, SP and scenario were not entered together as conventional fixed effects in the multivariable model; SP consistency was instead examined descriptively within each scenario. Because SP identity and patient sex were structurally linked to scenario in this design (two SPs per scenario, with the semaglutide scenario portrayed by women and the empagliflozin and apixaban scenarios portrayed by men), a cross-classified or random-intercept model cannot identify independent SP-, sex-, and scenario-level effects from these data [34]. Scenario contrasts are therefore interpreted as comparisons of whole standardized case packages rather than as pure drug, sex, or SP effects.

### 2.7. Visit Procedure, Reporting Probe, and Post-Encounter Debriefing

SPs visited during ordinary opening hours without advance contact. Immediately before entry, the SP recorded date, time, governorate, ownership type, whether a private or semi-private consultation area was visible, and workload, defined by the number of customers waiting for pharmacist attention. The interaction timer began with the opening request and ended when the consultation concluded. SPs did not request or purchase a prescription medicine. If the pharmacist recommended an over-the-counter product, the SP deferred purchase using the standardized exit statement. To reduce contamination after debriefing, daily routes were geographically dispersed where feasible, route lists were concealed from SPs until release, and no more than one branch of the same chain was assigned to the same SP on a single day. Debriefers did not disclose future pharmacy locations, route timing, or the identities of other SPs. A prespecified emergency-safety rule prevented unnecessary use of emergency resources: if a pharmacist attempted to call an ambulance or emergency service, the SP stated that family transport had already been arranged; if an emergency call was nevertheless initiated, the study was disclosed immediately before dispatch. No emergency medical service was activated because of a study encounter.

The pharmacist first responded without prompting. All clinical assessment, recognition, triage, urgency, medication-advice, and unsafe-management outcomes were closed for scoring when the pharmacist’s initial clinical advice was complete. If reportability had not been mentioned by that point, the SP then used one standardized reporting-readiness probe: ‘Could this be a reaction to my medicine, and is it something you can help report?’ The SP did not name the JFDA, suggest a website or application, or request a specific form. If the pharmacist attempted to submit a report, the SP allowed the pharmacist to identify the route and begin the information-gathering process, then stopped the process before transmission by stating that medication and contact details would be confirmed at home. No report based on a study encounter was submitted to the regulator, a manufacturer, or any other safety database.

Within 10 min of leaving, the SP completed a time-stamped electronic checklist and a short free-text reconstruction of key statements. The SP then returned to the pharmacy, disclosed the study, confirmed that the respondent was a licensed pharmacist, explained confidentiality, and sought verbal permission to retain and use the encounter data. Pharmacists who declined were thanked and their records were permanently removed before analysis. Post-encounter consent therefore allowed participants to refuse retention and research use of the covert-observation data; it did not imply that the prior observation itself could be undone. The debrief included a concise safety explanation and the official JFDA reporting route, deliberately withheld individual performance scoring, and asked pharmacists not to circulate scenario details until fieldwork was complete. At debriefing, the pharmacist was asked whether the visit had been suspected. Confirmed detection or material departure from the script triggered exclusion according to the protocol.

### 2.8. Data-Collection Instrument and Quality Assurance

The checklist contained four modules: pharmacy and visit context; information gathering; recognition and management; and pharmacovigilance behavior. Most items were binary and behaviorally anchored—for example, “explicitly advised emergency assessment now/today” rather than “gave good referral advice.” Medication-related statements were captured verbatim where possible and then coded. A formative checklist was used because items represented distinct safety behaviors and were not assumed to reflect a single latent construct; internal-consistency statistics were therefore not calculated. The operational scoring definitions are provided in Table S1. Because the checklist was formative—its items were clinically distinct components that jointly defined performance rather than interchangeable manifestations of a single latent trait—latent-trait approaches such as item-response theory were not considered appropriate for this measurement structure [35].

The electronic form used range checks, mandatory primary-outcome fields, automated timestamps, and unique visit codes. A field supervisor reviewed every submission on the day of collection for contradictions between the checklist and narrative note. Queries were resolved with the SP before the next route. A blinded second researcher independently coded all free-text statements and a random 15% sample of complete encounters. Agreement on the primary outcome was 95.7% (κ = 0.91). Remaining disagreements were resolved by consensus without knowledge of pharmacy identity. No audio or video was recorded during live encounters to protect privacy and preserve naturalistic conditions.

### 2.9. Outcome Definitions

The primary outcome was a guideline-informed safe-triage response. An encounter met the definition only when all three conditions were documented during the pre-probe clinical segment: (1) referral to an emergency department or emergency medical service; (2) explicit communication that assessment was immediate, urgent, or required the same day; and (3) no reassurance, “wait and see” advice, or product recommendation framed as a substitute for urgent assessment. A product offered solely for comfort while the pharmacist maintained immediate referral did not automatically fail the outcome; adjudication focused on whether the recommendation could reasonably delay escalation.

Secondary clinical outcomes were inquiry into individual assessment domains; adequate assessment, defined as four or more of six core domains (suspected medicine and dose, treatment or dose-change timing, symptom onset and course, severity or red flags, concomitant medicines, and relevant medical history); recognition of a possible medicine-related cause; naming the scenario-specific syndrome or harm; urgent referral; explicit urgency; and unsafe management likely to delay care. For the combined medicine-and-dose domain, both identification of the suspected product and ascertainment/confirmation of its dose were required; medicine identity and dose are nevertheless reported separately in the results section. The ≥4/6 assessment threshold was retained as a pragmatic majority-of-domains standard endorsed by the expert panel. Appropriate scenario-specific medicine advice was analyzed as a descriptive process measure because the required action differed by scenario: withhold the next semaglutide dose pending urgent review; temporarily withhold empagliflozin pending emergency assessment; or avoid further NSAIDs and avoid independent alteration of apixaban outside urgent medical instruction. These descriptive results are provided in Table S3. Behavioral anchors are provided in Table S1.

Clinical outcomes were coded exclusively from the unprompted pre-probe portion of the encounter; statements made only after the reporting-readiness probe could not retroactively satisfy medication-related recognition, syndrome recognition, referral, urgency, or medicine-advice outcomes. Pharmacovigilance outcomes formed two explicitly separated stages. Spontaneous reportability meant that the pharmacist raised ADR reporting before any reporting probe. For encounters without spontaneous reporting discussion, prompted reportability meant that the pharmacist agreed after the standardized probe that the case should or could be reported. Naming a legitimate route required identification of an official JFDA/WHO-UMC electronic route, VigiMobile, or another legitimate pharmacovigilance channel capable of forwarding the case to the national system, such as manufacturer pharmacovigilance. Initiating reporting assistance required an observable action: opening an official interface, providing a direct official link or QR code, contacting a pharmacovigilance service, or beginning to collect minimum case elements with a stated intention to report. Accessing an official JFDA reporting interface was recorded separately. Minimum case-element elicitation required all four elements specified in the 2014 JFDA Individual Case Safety Report guidance used for pre-fieldwork instrument development: an identifiable patient, an identifiable reporter, a suspected product, and a suspected reaction [28]. The later Version 2.0 JFDA guidance [36], published for public consultation in 2026, is cited in the Discussion as updated JFDA pharmacovigilance context and did not alter the prespecified four-element scoring rule. General advice to “tell your doctor” did not meet a reporting outcome. Table S1 summarizes the scoring rubric and the separation of pre-probe clinical outcomes from post-probe reporting-readiness outcomes.

### 2.10. Explanatory Variables

Explanatory variables entered in the multivariable model were scenario, governorate, independent versus chain ownership, visible consultation area, high workload, daytime versus evening visit, pharmacist gender, and self-reported formal pharmacovigilance training during the preceding two years. High workload was defined a priori as three or more customers waiting for pharmacist attention; this pragmatic threshold was retained after pilot work showed that queues of this size were the point at which SPs most consistently observed divided pharmacist attention and shortened interaction. Urbanicity was not included because all study pharmacies were urban by design. Highest qualification and years in practice were summarized descriptively but were not included in the primary model because they were collected after performance observation and had greater potential for nonresponse or misclassification. Recent pharmacovigilance training was also collected during debriefing and was therefore treated as a self-reported explanatory variable rather than an objectively verified exposure.

### 2.11. Ethical Considerations

The protocol was reviewed and approved by the Institutional Review Board of Al-Ahliyya Amman University before the study commenced (AAU/PHARM/2/4/2/2025–2026; approved 25 November 2025). The committee approved a waiver of prior consent because disclosure before the visit would invalidate measurement of routine behavior and because the encounter presented minimal risk, involved no real patient, required no medicine purchase, prohibited submission of a false safety report, and incorporated an emergency-resource protection rule. Immediate debriefing and post-encounter verbal consent allowed pharmacists to refuse retention and research use of their encounter data; data were destroyed when consent was declined. Pharmacy names, pharmacist names, precise addresses, and licensing numbers were not retained in the analytic dataset. The linkage file used for route management was encrypted, accessible only to the field coordinator, and deleted after completion of debriefing and quality checks. Results were analyzed and reported only in aggregate. Because precise pharmacy locations were intentionally not retained in the analytic dataset and the route-management linkage file was deleted after fieldwork quality checks, post hoc coordinate-based spatial autocorrelation analyses could not be undertaken within the approved data-governance framework.

### 2.12. Statistical Analysis

Analyses were conducted using IBM SPSS Statistics version 29.0 (IBM Corp., Armonk, NY, USA) and R version 4.5.0 (R Foundation for Statistical Computing, Vienna, Austria). In R, design-based estimates incorporating stratification, sampling weights, and finite-population correction were obtained using the survey package; robust and organization-clustered sandwich covariance estimates were calculated using the sandwich package; and multicollinearity diagnostics were obtained using the car package. Categorical variables are reported as number and percentage; interaction duration and years in practice are reported as median and interquartile range (IQR). For the overall primary prevalence estimate, the 95% confidence interval incorporated the stratified sampling design, governorate frame weights, and finite-population correction using the known eligible-frame totals. An uncorrected Wilson score interval was also calculated as a sensitivity estimate [37]. Because scenario assignment was randomized within governorate strata, pharmacy and pharmacist characteristics by scenario were presented descriptively without null-hypothesis tests of baseline balance. Pearson χ^2^ tests compared categorical outcomes across scenarios; when expected cell counts were sparse in a 3 × 2 table, the Fisher–Freeman–Halton exact test was used. All tests were two-sided.

The overall scenario comparison for the primary safe-triage outcome used α = 0.05. Pairwise primary-outcome comparisons used two-sided Fisher exact tests with Holm adjustment. For multiplicity control among secondary outcomes, the nine information-gathering outcomes in and the ten full-denominator clinical/pharmacovigilance outcomes were treated as two conceptually distinct analysis families and evaluated using Benjamini–Hochberg false-discovery-rate control at 5%; raw *p* values and *q* values are reported. The primary safe-triage outcome was not included in either FDR family. Conditional rows among pharmacists who spontaneously discussed reporting or among the 352 encounters receiving the reporting probe were descriptive only, because those denominators were defined by post-response behavior. For selected clinically important scenario contrasts—safe triage, medication-related recognition, specific syndrome/harm recognition, and unsafe management—we additionally estimated unadjusted prevalence ratios (PRs) and absolute prevalence differences with 95% confidence intervals. Required outcome fields were complete, and no imputation was performed.

Because safe triage was common, the multivariable association model used modified Poisson regression with a log link and robust sandwich standard errors to estimate adjusted prevalence ratios (aPRs) and 95% confidence intervals rather than odds ratios [38]. Scenario, governorate, ownership, consultation area, workload, visit period, pharmacist gender, and recent formal pharmacovigilance training were entered simultaneously; no univariable screening or stepwise selection was used. Reference categories were the semaglutide scenario, independent pharmacy, low/moderate workload, no visible consultation area, no recent training, male pharmacist, Amman governorate, and daytime. Multicollinearity was assessed using generalized variance-inflation factors. Because scenario and governorate were multi-degree-of-freedom factors, the adjusted measure GVIF ^(1/(2×Df))^ was used for comparability across terms; the maximum adjusted GVIF was 1.07, indicating no evidence of problematic multicollinearity. SP was not entered with scenario because the two SPs assigned to each scenario were structurally nested within that scenario; SP-specific descriptive results are therefore provided in Table S2 rather than modeled as an independent scenario-adjusted effect. To examine within-organization correlation, the identical modified Poisson model was re-estimated with sandwich covariance clustered by pharmacy organization; the 287 independent pharmacies were treated as singleton clusters and the 97 chain branches were grouped within 11 chain organizations, yielding 298 clusters. Organization-clustered results are reported in Table S5. To examine the maximum possible impact of the 19 post-encounter consent refusals on the overall primary prevalence, we additionally calculated deterministic extreme-case bounds among the 403 otherwise retainable completed encounters: all 19 refusals were first assigned an unsafe-triage outcome and then a safe-triage outcome.

The multivariable model was prespecified to estimate adjusted associations rather than causal effects or individual-level predictions. Accordingly, inverse-probability weighting or targeted maximum-likelihood estimation was not applied: these estimators still require causal-identification assumptions, including conditional exchangeability/no unmeasured confounding, that were not established by the present observational design [39]. Causal mediation and structural-equation analyses were likewise not undertaken because no causal mediation structure, temporal mediator sequence, or corresponding identification assumptions were prespecified [40]. Post hoc observed-power calculations were not performed because observed power is determined by the observed test result and does not add information beyond effect estimates, *p* values, and confidence intervals [41]. Because the modified-Poisson model used robust sandwich variance estimation, inference did not rely on the conventional Poisson equidispersion assumption [38]. AIC/BIC-based model selection and prediction-performance measures such as the Brier score or calibration assessment were not used because the covariate set was prespecified for association estimation rather than automated model selection or clinical prediction [42]. No post hoc interaction terms, nonlinear workload splines, or alternative ordinal definitions of the primary outcome were introduced after inspection of the results; the prespecified binary safe-triage definition and workload classification were retained.

## 3. Results

### 3.1. Study Flow and Encounter Characteristics

**Figure 1:**
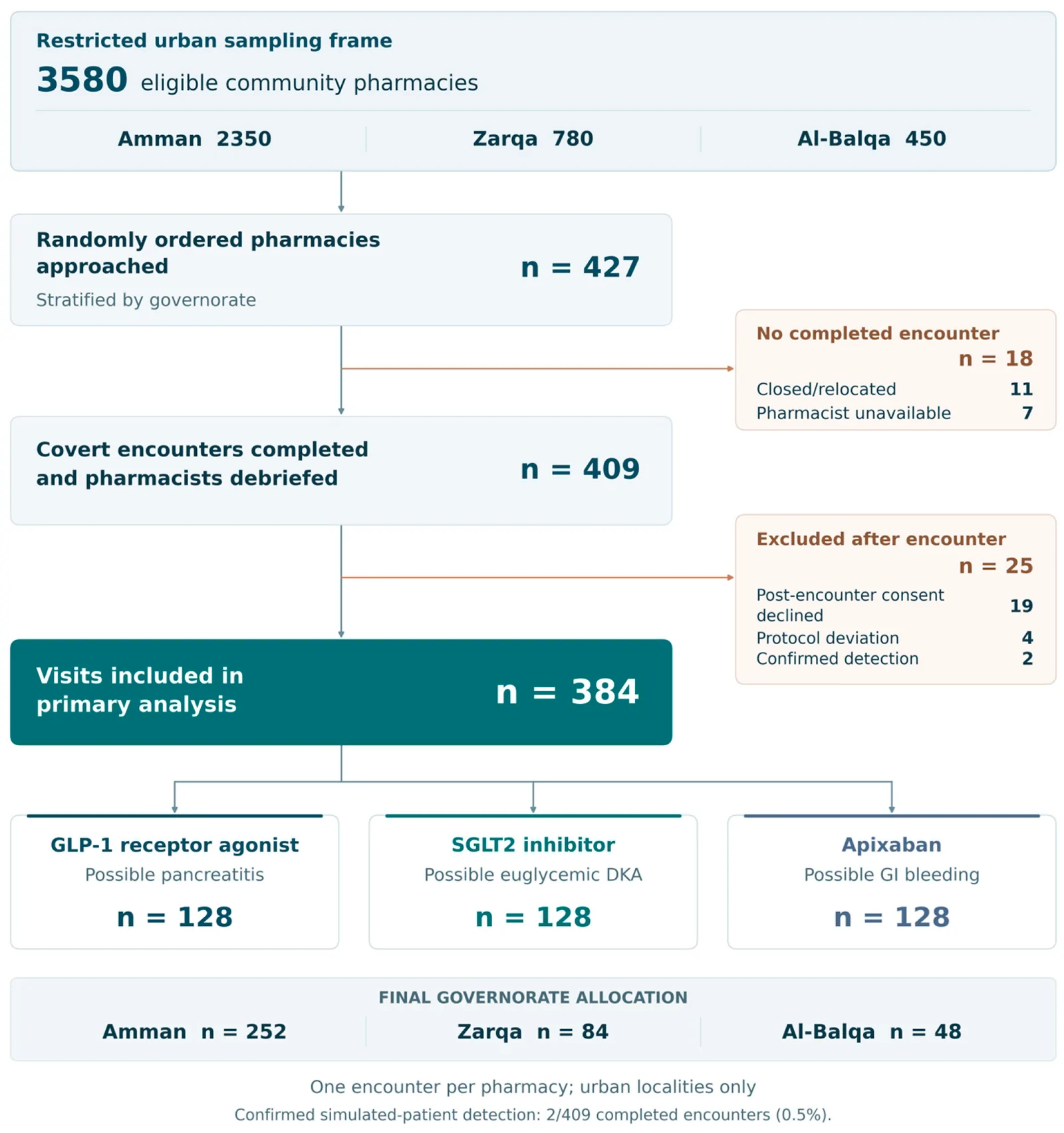
Flow of the restricted urban sampling frame, pharmacy approaches, post-encounter exclusions, and scenario allocation. Confirmed simulated-patient detection accounted for 2/409 completed encounters (0.5%).

Of 427 pharmacies approached, 409 completed encounters and 384 were retained after post-encounter consent and protocol/detection exclusions (Figure 1). The final sample comprised 128 encounters per scenario, with 252 visits in Amman, 84 in Zarqa, and 48 in Al-Balqa.

All sampled pharmacies were urban by design. Ninety-seven encounters occurred in chain branches belonging to 11 distinct chain organizations; the remaining 287 were independent pharmacies. Pharmacists and visits covered a range of practice experience, workload conditions, consultation environments, and visit periods. Full characteristics are shown in Table 1.

### 3.2. Clinical Assessment and Information Gathering

Information gathering was strongest for suspected-medicine identification (76.6%) and symptom onset/duration (69.0%). Dose was established in 40.6%, concomitant medicines in 42.4%, relevant history/risk factors in 46.6%, and allergy or prior ADR history in 15.4%. Overall, 31.0% met the ≥4/6 adequate-assessment threshold. After FDR correction, scenario differences remained for dose ascertainment, treatment/dose-change timing, and red-flag assessment, whereas the difference in adequate assessment did not remain significant (Table 2).

The strongest scenario contrast in information gathering concerned treatment timing and red-flag assessment. Treatment or dose-change timing was established in 59.4% of semaglutide encounters, 56.2% of empagliflozin encounters, and 30.5% of apixaban encounters; red flags were assessed in 53.1%, 43.0%, and 70.3%, respectively. These domain-level results are directly reported in Table 2.

**Table 2:** Information-gathering behavior overall and by adverse drug reaction scenario (N = 384).

Assessment behavior	Overall (N = 384)	GLP-1 RA (n = 128)	SGLT2 Inhibitor (n = 128)	Apixaban (n = 128)	Raw *p*	*q* Value
Asked medicine identity	294 (76.6)	101 (78.9)	92 (71.9)	101 (78.9)	0.309	0.556
Asked dose	156 (40.6)	65 (50.8)	43 (33.6)	48 (37.5)	0.013	0.040
Asked initiation/dose-change timing	187 (48.7)	76 (59.4)	72 (56.2)	39 (30.5)	<0.001	<0.001
Asked symptom onset/duration	265 (69.0)	85 (66.4)	90 (70.3)	90 (70.3)	0.738	0.830
Assessed severity/red flags	213 (55.5)	68 (53.1)	55 (43.0)	90 (70.3)	<0.001	<0.001
Asked concomitant medicines	163 (42.4)	54 (42.2)	53 (41.4)	56 (43.8)	0.928	0.928
Asked relevant history/risk factors	179 (46.6)	50 (39.1)	58 (45.3)	71 (55.5)	0.029	0.066
Asked allergy or prior ADR	59 (15.4)	22 (17.2)	17 (13.3)	20 (15.6)	0.684	0.830
Adequate assessment (≥4/6 domains)	119 (31.0)	45 (35.2)	39 (30.5)	35 (27.3)	0.396	0.595

Note: Values are n (%). Raw *p* values compare the three standardized scenarios. *q* values are Benjamini–Hochberg FDR-adjusted across the nine information-gathering outcomes shown in this table. ADR, adverse drug reaction; RA, receptor agonist; SGLT2, sodium-glucose cotransporter-2. The six-domain adequate-assessment composite maps the displayed items as follows: the medicine-and-dose domain requires both medicine identity and dose; treatment timing, symptom onset/course, severity/red flags, concomitant medicines, and relevant history/risk factors each constitute one domain. Allergy/prior ADR is reported separately and is not included in the six-domain composite.

### 3.3. Recognition and Safe Triage of Suspected Serious ADRs

A possible medication-related cause was recognized before the reporting probe in 72.9% of encounters, whereas the specific syndrome or harm was named in 39.6%. Recognition was highest in the apixaban scenario and lowest in the empagliflozin scenario; these differences remained significant after FDR control (Table 3).

Safe triage, defined as ED/EMS referral plus explicit urgency plus no delay-promoting management, occurred in 41.7% of encounters (design-based 95% CI, 37.0–46.3; Wilson sensitivity interval, 36.8–46.7). Performance differed by scenario: 60.9% for apixaban, 42.2% for semaglutide, and 21.9% for empagliflozin (overall *p* < 0.001). Holm-adjusted pairwise *p* values were 0.002 for empagliflozin versus semaglutide, 0.004 for apixaban versus semaglutide, and <0.001 for apixaban versus empagliflozin. Pairwise effect estimates are shown in Table 4.

In the extreme-case consent sensitivity analysis, the overall safe-triage prevalence would range from 39.7% (160/403 if all 19 consent decliners had failed safe triage) to 44.4% (179/403 if all 19 had achieved safe triage); thus, even the most favorable extreme assignment remained below 50% (Table S6).

Unsafe management likely to delay assessment occurred in 26.3% of encounters and was most frequent in the empagliflozin scenario (40.6%) and least frequent in the apixaban scenario (13.3%). Commonly coded delaying behaviors included symptomatic product recommendations positioned as an alternative to urgent assessment, wait-and-see advice, or reassurance that reduced the immediacy of referral. Scenario-specific medicine advice is reported descriptively in Table S3 rather than included in the cross-scenario inferential family because the required action differed by scenario. Appropriate scenario-specific medicine advice was provided in 67/128 (52.3%) semaglutide encounters, 54/128 (42.2%) empagliflozin encounters, and 82/128 (64.1%) apixaban encounters; notably, temporary withholding of empagliflozin pending emergency assessment was advised in only 42.2% of SGLT2-inhibitor encounters (Table S3).

**Table 3:** Recognition, triage, unsafe management, and pharmacovigilance behavior by scenario (N = 384).

Outcome	Overall (N = 384)	GLP-1 RA (n = 128)	SGLT2 Inhibitor (n = 128)	Apixaban (n = 128)	Raw *p*	*q* Value
Medication-related cause recognized (pre-probe)	280 (72.9)	90 (70.3)	70 (54.7)	120 (93.8)	<0.001	<0.001
Specific syndrome/harm named (pre-probe)	152 (39.6)	41 (32.0)	23 (18.0)	88 (68.8)	<0.001	<0.001
Urgent ED/EMS referral (pre-probe)	250 (65.1)	83 (64.8)	68 (53.1)	99 (77.3)	<0.001	<0.001
Urgency explicitly communicated (pre-probe)	202 (52.6)	68 (53.1)	45 (35.2)	89 (69.5)	<0.001	<0.001
Primary: safe triage response (pre-probe)	160 (41.7)	54 (42.2)	28 (21.9)	78 (60.9)	<0.001	—
Unsafe management likely to delay care (pre-probe)	101 (26.3)	32 (25.0)	52 (40.6)	17 (13.3)	<0.001	<0.001
Spontaneously identified as reportable (pre-probe)	32 (8.3)	14 (10.9)	8 (6.2)	10 (7.8)	0.385	0.481
Among spontaneous-reporting encounters: initiated reporting assistance before probe	18/32 (56.2)	8/14 (57.1)	4/8 (50.0)	6/10 (60.0)	—	—
Among probed encounters: recognized as reportable after probe	115/352 (32.7)	39/114 (34.2)	33/120 (27.5)	43/118 (36.4)	—	—
Among probed encounters: initiated reporting assistance after probe	28/352 (8.0)	10/114 (8.8)	7/120 (5.8)	11/118 (9.3)	—	—
Named legitimate reporting route by end of encounter	58 (15.1)	23 (18.0)	14 (10.9)	21 (16.4)	0.256	0.427
Initiated reporting assistance by end of encounter	46 (12.0)	18 (14.1)	11 (8.6)	17 (13.3)	0.346	0.481
Accessed official JFDA reporting interface	11 (2.9)	2 (1.6)	5 (3.9)	4 (3.1)	0.640	0.711
Elicited four minimum case elements by end of encounter	23 (6.0)	9 (7.0)	6 (4.7)	8 (6.2)	0.723	0.723

Note: Values are n (%) unless a row-specific denominator is shown. The primary safe-triage outcome requires ED/EMS referral, explicit urgency, and no delay-promoting management. Clinical outcomes were scored before the reporting-readiness probe. Conditional rows among the 32 spontaneous-reporting encounters or the 352 probed encounters are descriptive only. For the ten full-denominator secondary outcomes, raw *p* values and Benjamini–Hochberg *q* values are shown; the primary outcome is analyzed separately and is not included in the FDR family. ED, emergency department; EMS, emergency medical service; JFDA, Jordan Food and Drug Administration; RA, receptor agonist; SGLT2, sodium-glucose cotransporter-2.

**Table 4:** Pairwise effect estimates for selected scenario-level outcomes (unadjusted).

Outcome	Scenario Contrast	Prevalence Ratio (95% CI)	Absolute Difference, Percentage Points (95% CI)
Safe triage	SGLT2 inhibitor vs. GLP-1 RA	0.52 (0.35–0.76) Holm *p* 0.002	−20.3 (−31.0 to −8.9)
Safe triage	Apixaban vs. GLP-1 RA	1.44 (1.13–1.85) Holm *p* 0.004	+18.8 (+6.5 to +30.2)
Safe triage	Apixaban vs. SGLT2 inhibitor	2.79 (1.95–3.98) Holm *p* < 0.001	+39.1 (+27.3 to +49.2)
Medication-related recognition	SGLT2 inhibitor vs. GLP-1 RA	0.78 (0.64–0.94)	−15.6 (−26.9 to −3.8)
Medication-related recognition	Apixaban vs. GLP-1 RA	1.33 (1.18–1.51)	+23.4 (+14.3 to +32.4)
Medication-related recognition	Apixaban vs. SGLT2 inhibitor	1.71 (1.46–2.02)	+39.1 (+29.0 to +48.2)
Specific syndrome/harm named	SGLT2 inhibitor vs. GLP-1 RA	0.56 (0.36–0.88)	−14.1 (−24.3 to −3.4)
Specific syndrome/harm named	Apixaban vs. GLP-1 RA	2.15 (1.63–2.83)	+36.7 (+24.7 to +47.2)
Specific syndrome/harm named	Apixaban vs. SGLT2 inhibitor	3.83 (2.60–5.64)	+50.8 (+39.4 to +60.1)
Unsafe management	SGLT2 inhibitor vs. GLP-1 RA	1.62 (1.13–2.34)	+15.6 (+4.1 to +26.6)
Unsafe management	Apixaban vs. GLP-1 RA	0.53 (0.31–0.91)	−11.7 (−21.2 to −2.1)
Unsafe management	Apixaban vs. SGLT2 inhibitor	0.33 (0.20–0.53)	−27.3 (−37.3 to −16.7)

Note: PRs compare prevalence in the first-listed scenario with the second-listed scenario. Absolute differences are first minus second. PR confidence intervals use log-scale standard errors; absolute-difference confidence intervals use the Newcombe method. Holm-adjusted *p* values are shown for the three primary safe-triage pairwise comparisons only. Other rows are presented as effect-size estimates without additional pairwise multiplicity testing.

### 3.4. Pharmacovigilance Reporting Behavior

Only 8.3% of pharmacists spontaneously raised ADR reporting before the standardized probe. Among the 352 encounters receiving the probe, 32.7% subsequently regarded the case as reportable and 8.0% initiated observable reporting assistance. By the end of the encounter, 12.0% had initiated reporting assistance, 15.1% had named a legitimate reporting route, and 2.9% had accessed an official JFDA reporting interface. These post-probe outcomes represent prompted reporting readiness rather than spontaneous reporting behavior (Table 3).

**Figure 2:**
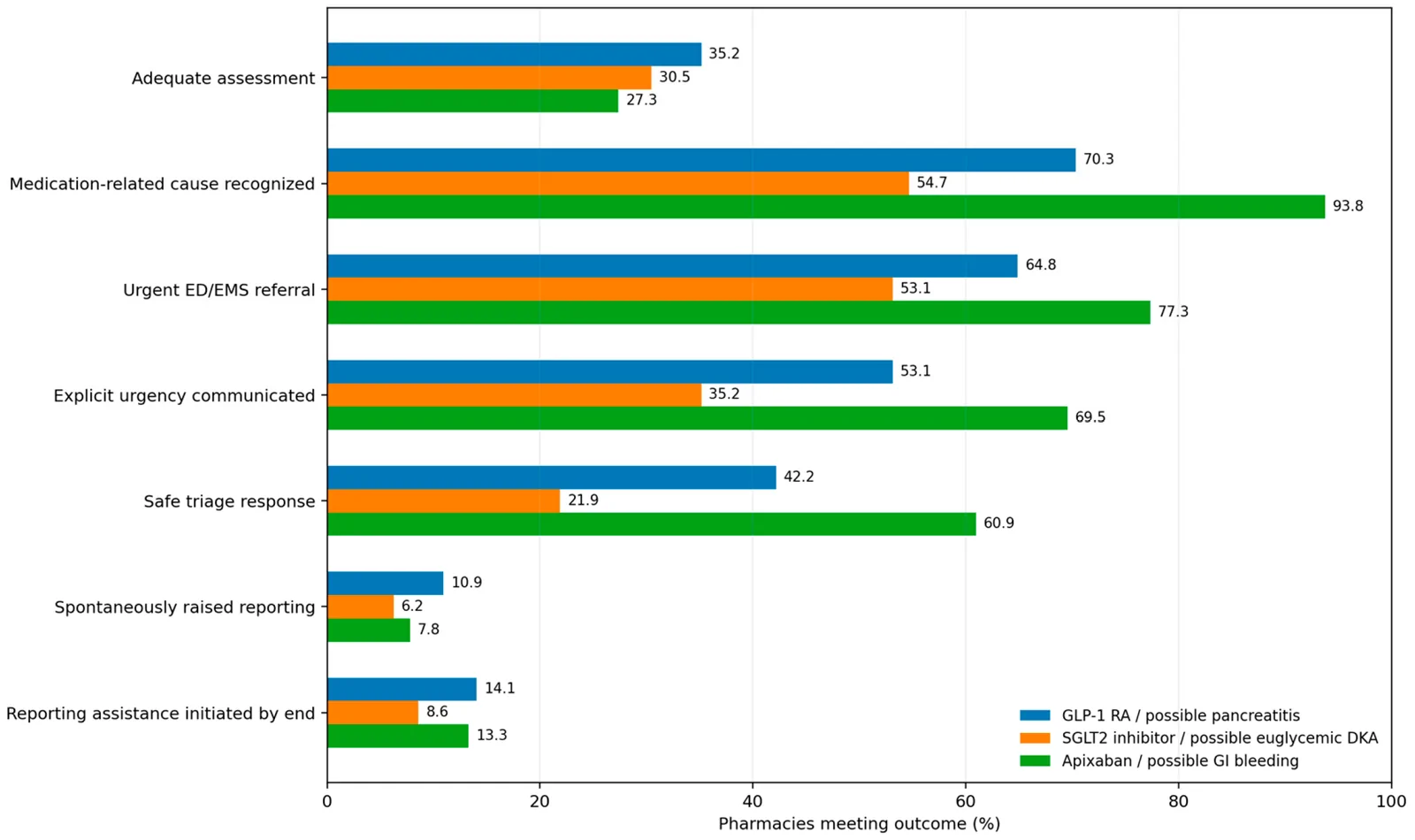
Selected clinical and pharmacovigilance outcomes by standardized scenario. Clinical outcomes and spontaneous reporting were measured before the reporting-readiness probe. “Reporting assistance initiated by end of encounter” combines actions initiated spontaneously and actions initiated after the standardized probe. Outcomes are displayed side by side and should not be interpreted as sequentially nested stages.

Observable reporting action remained uncommon even within clinically stronger consultations: reporting assistance had been initiated by the end of the encounter in 38/280 (13.6%) encounters in which a medication-related cause was recognized and in 26/160 (16.3%) encounters meeting the safe-triage definition. The corresponding proportions were 8/104 (7.7%) when a medication-related cause was not recognized and 20/224 (8.9%) when safe triage was not achieved. These subgroup cross-tabulations are presented in Table S4. Figure 2 displays selected clinical and pharmacovigilance outcomes by standardized scenario without implying that the outcomes form a mathematically nested cascade.

### 3.5. Factors Associated with Safe Triage

In the modified Poisson model, safe triage was less prevalent in the empagliflozin scenario than in the semaglutide reference scenario (aPR, 0.51; 95% CI, 0.35–0.74) and more prevalent in the apixaban scenario (aPR, 1.54; 95% CI, 1.21–1.96). High workload was associated with lower safe-triage prevalence (aPR, 0.53; 95% CI, 0.39–0.72), whereas a visible consultation area (aPR, 1.32; 95% CI, 1.05–1.67) and recent formal pharmacovigilance training (aPR, 1.33; 95% CI, 1.01–1.74) were associated with higher prevalence. Chain ownership, pharmacist gender, governorate, and visit period were not independently associated with safe triage (Table 5).

SP-specific descriptive checks supported the direction of the scenario-level findings. Within a scenario, the two SPs differed in safe-triage prevalence by 3.1–9.4 percentage points; medication-related recognition differed by 3.1–12.5 points and syndrome-specific recognition by 6.3–10.9 points (Table S2). Because each SP remained nested within one scenario, these checks cannot identify an SP-independent causal scenario effect. In the organization-clustered sensitivity analysis, regression coefficients were unchanged, as expected when only the covariance estimator is changed, and confidence intervals/*p* values were very similar to the primary robust analysis (Table S5). These adjusted estimates are interpreted as associations and do not establish causal effects of workload, consultation space, training, ownership, or other explanatory variables.

## 4. Discussion

### 4.1. Principal Clinical Findings

This behavior-based assessment identifies a clinically important gap in first-contact management of suspected serious ADRs. Only 41.7% of encounters met all three safe-triage requirements: ED/EMS referral, explicit communication of urgency, and absence of delay-promoting management. Performance differed markedly across the standardized scenario packages: apixaban-associated gastrointestinal bleeding was most consistently recognized and escalated, semaglutide-associated pancreatitis showed intermediate performance, and the euglycemic empagliflozin presentation was most frequently missed. These findings reinforce that medication-related recognition, syndrome identification, referral, explicit urgency communication, and avoidance of delaying advice are related but distinct clinical behaviors. Pharmacovigilance engagement represented an additional implementation gap, with spontaneous reporting discussion and observable reporting action remaining uncommon.

### 4.2. Clinical Recognition and Triage Across Three Serious ADR Presentations

The strongest performance occurred in the apixaban scenario, in which black stools and dizziness during anticoagulant therapy provided salient bleeding cues and recent ibuprofen use increased concern. Even so, 39.1% of encounters failed the strict safe-triage definition and 13.3% included management likely to delay care. This residual gap is clinically relevant because patients may initially interpret melena, weakness, or dizziness as a minor gastrointestinal complaint and seek pharmacy advice before emergency assessment.

The semaglutide scenario illustrates the difficulty of distinguishing common gastrointestinal adverse effects from a potentially serious medicine-related event. Severe persistent epigastric pain radiating to the back after dose escalation should raise concern for acute pancreatitis and prompt urgent assessment. Semaglutide product information available before fieldwork identified acute pancreatitis as a recognized risk and advised discontinuation when pancreatitis is suspected [30]. In this analysis, 32.0% named the specific syndrome, 42.2% achieved strict safe triage, and 25.0% received management that could delay assessment. Training should therefore emphasize red-flag patterns, escalation, and explicit urgency rather than undifferentiated lists of adverse effects.

The empagliflozin scenario produced the weakest clinical performance. A glucose value of 165 mg/dL may falsely reassure clinicians who associate ketoacidosis with marked hyperglycemia, although regulators warn that SGLT2 inhibitor-associated ketoacidosis can occur without very high glucose [29]. Only 18.0% named the specific syndrome, 21.9% met the strict safe-triage definition, and 40.6% received potentially delaying management. The combination of deep breathing, abdominal symptoms, reduced intake, and intercurrent illness therefore represents a focused learning opportunity around sick-day management and euglycemic ketoacidosis.

### 4.3. Clinical Management and Pharmacovigilance as Distinct Safety Behaviors

The second major finding was limited spontaneous pharmacovigilance engagement. Only 8.3% of pharmacists raised reporting before any prompt. The standardized probe explicitly asked whether the symptoms could represent a medicine reaction and whether the pharmacist could help report it; post-probe responses therefore reflect reporting readiness under a direct patient cue rather than naturalistic spontaneous behavior. Among probed encounters, 32.7% acknowledged reportability and 8.0% initiated observable reporting assistance. JFDA materials accept suspected ADR reports from healthcare professionals and define the minimum elements of a valid individual case safety report [9,28,36]. Definitive causal attribution is therefore not required before a clinically credible suspected serious ADR can enter the reporting pathway.

This pattern is consistent with, but extends, Jordanian and regional self-report evidence. Jordanian surveys and qualitative work have documented incomplete knowledge, low reporting engagement, time pressure, procedural complexity, and absent feedback [13,14,15,16]. A Saudi community-pharmacist survey similarly identified limited knowledge of the responsible pharmacovigilance center and lack of time as barriers [7], while an educational study among Yemeni community pharmacists improved pharmacovigilance knowledge without establishing sustained real-world reporting behavior [8]. The present behavioral assessment suggests that even relatively strong clinical encounters do not automatically translate into observable reporting action; however, reporting during an acute encounter should remain secondary to protecting the patient and arranging urgent assessment.

Reporting should therefore be viewed as a complementary step in medication safety rather than as a substitute for immediate clinical action. In a serious presentation, urgent referral appropriately takes priority; however, once the patient is protected, a reporting process that depends on memory, navigation through unfamiliar websites, or lengthy data entry may be abandoned. This distinction suggests that interventions should protect the clinical encounter first and make reporting a low-burden continuation of care rather than a competing task.

Responsibility for pharmacovigilance reporting should not be viewed as transferring exclusively from the community pharmacist to the emergency department after referral. Emergency clinicians and hospital pharmacovigilance systems may identify additional clinical information and can submit or complete a report, while the referring community pharmacist can also contribute a report based on the suspected medicine-related event observed at first contact. A shared reporting pathway, with immediate patient safety taking precedence and subsequent reporting supported across care transitions, is therefore preferable to assigning reporting to a single professional group.

### 4.4. Practice-Level Factors Associated with Safe Triage

High workload was associated with approximately half the adjusted prevalence of safe triage (aPR, 0.53). This association is clinically plausible because a strict safe-triage response requires recognition, focused questioning, a clear emergency recommendation, explicit timing language, and avoidance of symptom-oriented advice that could dilute the referral message. A visible consultation area was associated with higher safe-triage prevalence (aPR, 1.32), potentially reflecting greater privacy, more opportunity for focused assessment, or a broader clinical-service orientation. These observational associations identify implementation targets rather than causal effects.

Recent pharmacovigilance training was also associated with higher adjusted safe-triage prevalence (aPR, 1.33), although training was self-reported and the study cannot establish causality. Chain ownership was not independently associated with safe triage. Importantly, using organization-clustered covariance for the 11 chain organizations produced materially similar confidence intervals and did not alter the substantive interpretation (Table S5). These findings support prospective evaluation of structured medication-safety protocols, scenario-based training, and workflow interventions rather than attributing performance differences to ownership type itself.

### 4.5. Implications for Clinical Practice and Medication Safety

The immediate clinical implication is the need to strengthen recognition of drug-specific red flags and the language of escalation. Scenario-based continuing professional development could contrast common, self-limiting adverse effects with high-consequence patterns such as euglycemic ketoacidosis during SGLT2 inhibitor therapy, severe persistent abdominal pain during GLP-1 receptor agonist therapy, and bleeding during oral anticoagulation. Training should emphasize a simple sequence: suspect serious medicine-related harm, identify the key red flags, communicate urgency clearly, and avoid symptomatic advice that could delay definitive assessment.

A second target is workflow support for safe triage. Brief decision prompts embedded in pharmacy procedures could remind pharmacists to assess severity, recent treatment changes, interacting medicines, and high-risk symptom patterns before recommending a product. Protected consultation space and staffing arrangements may also merit evaluation, particularly during high-workload periods. Such approaches should be tested prospectively because the present study cannot determine whether these organizational features themselves cause better performance.

Pharmacovigilance integration should complement this clinical pathway. Once urgent care has been arranged, rapid access to existing JFDA digital reporting routes—including the electronic reporting interface and VigiMobile—could reduce the additional burden of converting a suspected ADR into a report [43]. A concise medication-safety prompt could link the clinical and surveillance steps: protect the patient, identify the suspected medicine, capture the minimum valid-case elements, and initiate the report. Reporter confirmation and structured feedback may further reinforce engagement without delaying emergency management.

These priorities align with the World Health Organization’s broader emphasis on reducing avoidable medication-related harm [44]. However, the observational design does not establish that any specific intervention will improve outcomes. Controlled implementation studies should therefore evaluate clinically meaningful endpoints—including correct recognition, explicit urgent referral, avoidance of delaying management, completed valid reports, and consultation burden—rather than knowledge scores alone.

### 4.6. Strengths and Limitations

The study has several strengths. It used a proportionately stratified probability sample drawn from a defined urban sampling frame across Amman, Zarqa, and Al-Balqa; measured behavior in routine community-pharmacy practice rather than intention; included three pharmacologically and diagnostically distinct serious presentations; used expert validation and pilot testing; standardized SP delivery and scoring; retained a strict three-component primary safe-triage outcome; and separated unprompted clinical performance from prompted reporting readiness by closing clinical scoring before the reporting probe. One encounter per pharmacy, concealed route release, geographically dispersed scheduling where feasible, and limited same-chain scheduling reduced detection and spillover risk. CRiSPHe and STROBE reporting, explicit scoring rules, design-based precision estimates, effect-size reporting, SP-specific descriptive checks, and organization-clustered sensitivity analysis strengthen reproducibility and interpretation. Supplementary Tables S1–S6 provide the scoring rubric and key robustness analyses.

Limitations should temper interpretation. First, the study was restricted to urban community pharmacies in Amman, Zarqa, and Al-Balqa; rural and semi-rural settings and other regions of Jordan were not sampled, so the estimates should not be interpreted as national prevalence. Second, each pharmacy was assessed once, and performance may reflect the on-duty pharmacist and momentary conditions. Third, standardized cases cannot reproduce the full ambiguity, emotion, measurements, and longitudinal information of real patients. Fourth, each scenario was delivered by only two SPs, and SP identity was completely nested within the scenario. Patient sex was also part of the scenario package: the semaglutide case was portrayed by a woman, whereas the empagliflozin and apixaban cases were portrayed by men. Consequently, observed scenario contrasts represent whole-case packages that jointly include medicine, symptoms, patient sex, and SP identity; they cannot be decomposed into a pure pharmacological, sex, or SP-independent causal effect. SP-specific descriptive results reduce, but do not eliminate, this concern. Fifth, live visits were not recorded; immediate electronic entry, verbatim reconstruction, audit procedures, and strong mock reliability reduce but cannot eliminate recall or classification error. The study did not deliberately manipulate apparent age, frailty, body habitus, or other patient characteristics beyond those specified in the scenarios, and it did not include a deliberately elderly-patient portrayal. Pharmacist responses may differ when similar symptoms are presented by older, frail, or otherwise visibly higher-risk patients, so this dimension of external validity should be examined in future SP studies.

Sixth, immediate debriefing could have produced spillover if a pharmacist alerted colleagues or nearby pharmacies despite route-concealment and confidentiality safeguards. Confirmed SP detection was uncommon (2/409 completed encounters, 0.5%), but unrecognized or undisclosed suspicion cannot be excluded. Seventh, post-encounter consent may have introduced selection bias because 19 pharmacists declined retention of their encounter data. Those records were permanently destroyed as required by the approved procedure, so their scenario, governorate, and performance distributions cannot be examined; the design-based confidence interval therefore quantifies sampling variability but does not account for this potential post-encounter selection mechanism. Eighth, the reporting probe was intentionally explicit, so post-probe outcomes represent prompted reporting readiness rather than spontaneous behavior, and reporting workflows were stopped before transmission. Ninth, workload, consultation area, training, ownership, and other explanatory-variable associations are observational. Although organization-clustered covariance produced similar inference, only 11 chain organizations were represented. Finally, medicine-safety communications and reporting systems evolve, so official routes, scripts, and scoring rules should be revalidated before replication. The added extreme-case bounds show that the headline prevalence conclusion is robust to the most optimistic or pessimistic assignment of the 19 consent refusals; however, because those records were permanently destroyed, pattern-mixture or other model-based MNAR analyses requiring their covariate or outcome information are not identifiable from the retained dataset. These design and data-governance constraints define the estimands and sensitivity analyses that are identifiable from the retained data; they should therefore be treated as limitations for future study design rather than as uncertainties that can be removed by additional post hoc modelling.

### 4.7. Priorities for Clinical and Implementation Research

Future work should test clinically actionable mechanisms rather than repeat prevalence measurement alone. Cluster-randomized or factorial implementation studies could compare drug-specific red-flag training, workflow prompts for urgent referral, point-of-care access to JFDA/VigiMobile reporting routes, and structured feedback, separately and in combination. Time-motion and post-debrief cognitive interviews could identify where clinical assessment or reporting fails without contaminating the initial behavioral observation. Broader replication should include northern and southern governorates and rural or semi-rural settings, while an expanded scenario bank could examine pediatric dosing harm, pregnancy and lactation, drug–drug interactions, medication errors, and other high-risk presentations relevant to first-contact medication safety. Repeated-measures or crossover SP designs could help separate within-pharmacist from between-pharmacist variation, provided that carryover, recognition, and contamination are minimized through appropriate spacing and concealment. Future protocols could also prespecify graded triage outcomes (for example, optimal, defensible-but-incomplete, and unsafe escalation) and model workload continuously when the study is specifically designed to estimate dose–response relationships. Finally, medication-safety pathways should be evaluated across the broader community-based care team, including community pharmacists, primary-care clinicians, emergency clinicians, and hospital pharmacovigilance services, rather than treating ADR recognition and reporting as the responsibility of a single profession.

## 5. Conclusions

In this covert assessment of urban community pharmacies in central Jordan, recognition and strict safe triage of suspected serious ADRs were inconsistent and varied across standardized clinical scenarios. The largest deficit occurred in the empagliflozin-associated euglycemic ketoacidosis presentation, whereas apixaban-associated gastrointestinal bleeding was more consistently recognized and escalated. Safe triage required not only emergency referral but also explicit communication of urgency and avoidance of advice likely to delay care. Spontaneous pharmacovigilance engagement was limited, even though some reporting activity emerged after a direct patient cue. These findings support prospective evaluation of drug-specific red-flag training, explicit escalation protocols, workflow conditions that protect focused assessment, and low-burden integration of reporting after immediate patient safety has been addressed. Geographic transferability should be evaluated before national prevalence claims are made.

## Figures and Tables

**Table 1:** Pharmacy, pharmacist, and visit characteristics overall and by scenario (N = 384).

Characteristic	Overall (*N* = 384)	GLP-1 RA (n = 128)	SGLT2 Inhibitor (n = 128)	Apixaban (n = 128)
Governorate—Amman	252 (65.6)	84 (65.6)	84 (65.6)	84 (65.6)
Governorate—Zarqa	84 (21.9)	28 (21.9)	28 (21.9)	28 (21.9)
Governorate—Al-Balqa	48 (12.5)	16 (12.5)	16 (12.5)	16 (12.5)
Independent ownership	287 (74.7)	92 (71.9)	97 (75.8)	98 (76.6)
Female pharmacist	218 (56.8)	67 (52.3)	78 (60.9)	73 (57.0)
BPharm as highest degree	283 (73.7)	102 (79.7)	87 (68.0)	94 (73.4)
PharmD as highest degree	80 (20.8)	21 (16.4)	33 (25.8)	26 (20.3)
Postgraduate degree	21 (5.5)	5 (3.9)	8 (6.2)	8 (6.2)
Formal PV training, past 2 years	69 (18.0)	25 (19.5)	27 (21.1)	17 (13.3)
High workload at encounter	119 (31.0)	38 (29.7)	35 (27.3)	46 (35.9)
Consultation area visible	87 (22.7)	28 (21.9)	30 (23.4)	29 (22.7)
Evening visit	141 (36.7)	37 (28.9)	52 (40.6)	52 (40.6)
Interaction duration, min, median (IQR)	3.9 (2.9–5.6)	4.3 (3.1–5.9)	4.0 (2.9–5.5)	3.7 (2.7–5.5)
Years in practice, median (IQR)	8 (5–13)	9 (5–13)	8 (5–12)	8 (6–12)

Note: Values are n (%) unless otherwise stated. Scenario groups were randomized within governorate strata; descriptive characteristics are shown without baseline significance tests. IQR, interquartile range; PV, pharmacovigilance; RA, receptor agonist; SGLT2, sodium-glucose cotransporter-2. BPharm, Bachelor of Pharmacy; PharmD, Doctor of Pharmacy.

**Table 5:** Modified Poisson regression for guideline-informed safe triage (N = 384).

Predictor	Safe Response, n/N (%)	Adjusted PR (95% CI)	*p* Value
Scenario			
GLP-1 RA	54/128 (42.2)	1.00 (reference)	—
SGLT2 inhibitor	28/128 (21.9)	0.51 (0.35–0.74)	<0.001
Apixaban	78/128 (60.9)	1.54 (1.21–1.96)	<0.001
Ownership			
Independent	117/287 (40.8)	1.00 (reference)	—
Chain	43/97 (44.3)	1.03 (0.81–1.30)	0.823
Workload			
Low/moderate	128/265 (48.3)	1.00 (reference)	—
High	32/119 (26.9)	0.53 (0.39–0.72)	<0.001
Consultation area			
Not visible	115/297 (38.7)	1.00 (reference)	—
Visible	45/87 (51.7)	1.32 (1.05–1.67)	0.018
Formal PV training in past 2 years			
No	125/315 (39.7)	1.00 (reference)	—
Yes	35/69 (50.7)	1.33 (1.01–1.74)	0.039
Pharmacist gender			
Male	70/166 (42.2)	1.00 (reference)	—
Female	90/218 (41.3)	1.04 (0.83–1.29)	0.760
Governorate—Amman	103/252 (40.9)	1.00 (reference)	—
Governorate—Zarqa	38/84 (45.2)	1.08 (0.84–1.39)	0.534
Governorate—Al-Balqa	19/48 (39.6)	0.95 (0.68–1.34)	0.786
Visit period			
Daytime	102/243 (42.0)	1.00 (reference)	—
Evening	58/141 (41.1)	0.97 (0.77–1.22)	0.779

Note: Safe triage requires ED/EMS referral, explicit communication of urgency, and no management likely to delay care. Adjusted simultaneously for all variables shown using modified Poisson regression with robust sandwich standard errors. Reference categories are stated in the table; Amman is the reference governorate. Organization-clustered covariance results are shown in Table S5. aPR, adjusted prevalence ratio; CI, confidence interval; PV, pharmacovigilance; RA, receptor agonist; SGLT2, sodium-glucose cotransporter-2.

## Data Availability

The data presented in this study are available from the corresponding author on reasonable request. The dataset is not publicly available because unrestricted release of record-level data could compromise the confidentiality of participating pharmacists and pharmacies. Any data sharing will be limited to appropriately de-identified data and will be subject to the conditions of the approved ethics protocol.

## Supplementary Materials

The following supporting information can be downloaded at: https://www.mdpi.com/article/10.3390/jcm15218652/s1, Supplementary Material S1: Standardized simulated-patient scripts and SP rules; Table S1: Operational definitions and scoring rubric; Table S2: Simulated patient-specific descriptive consistency checks; Table S3: Appropriate scenario-specific medicine advice (Descriptive process measure); Table S4: Reporting assistance Initiated by End of Encounter According to Clinical Performance; Table S5: Organization-clustered sensitivity analysis for the safe-triage model; Table S6: Extreme-case sensitivity bounds for post-encounter consent refusals.

## Abbreviations

The following abbreviations are used in this manuscript:

ADR	adverse drug reaction
aPR	adjusted prevalence ratio
BPharm	Bachelor of Pharmacy
CI	confidence interval
CRiSPHe	Checklist for Reporting Research Using a Simulated Patient Methodology in Health
DKA	diabetic ketoacidosis
ED	emergency department
EMS	emergency medical service
FDR	false-discovery rate
GI	gastrointestinal
GLP-1 RA	glucagon-like peptide-1 receptor agonist
IQR	interquartile range
JFDA	Jordan Food and Drug Administration
JPA	Jordan Pharmacists Association
NSAID	nonsteroidal anti-inflammatory drug
PharmD	Doctor of Pharmacy
PR	prevalence ratio
PV	pharmacovigilance
SGLT2	sodium-glucose cotransporter-2
SP	simulated patient
STROBE	Strengthening the Reporting of Observational Studies in Epidemiology
WHO-UMC.	World Health Organization Uppsala Monitoring Centre
