# Supplementary material for: Clinical Recognition, Safe Triage, and Pharmacovigilance Reporting Readiness for Suspected Serious Adverse Drug Reactions in Community Pharmacies: A Covert Simulated-Patient Study in Central Jordan
Source: J Clin Med. 2026 Nov 6;15(21):8652. doi: 10.3390/jcm15218652 (PMC13764172; doi:10.3390/jcm15218652)
Supplement: Supplementary file 1 [file jcm-15-08652-s001.zip › jcm-4620942-supplementary.pdf]

Supplementary Materials

Clinical Recognition, Safe Triage, and Pharmacovigilance Reporting Readiness for Suspected Serious Adverse Drug Reactions in Community Pharmacies: A Covert Simulated-Patient Study in Central Jordan

Zekrayat J.H. Merdas; Mohammad Abu Assab; Anas Abed*; Wala'a Al. Safadi; Zain Z. Zakaria; Zainab Zakaraya; Sireen Abdul Rahim Shilbayeh

Supplementary Material S1. Standardized Simulated-Patient Scripts and SP Rules

SP rules

- Use the exact opening statement. Do not volunteer medication details, risk factors, or red flags beyond the opening statement unless the pharmacist asks the corresponding question.
- Answer only what is asked. If a question is unclear, request clarification once; do not help the pharmacist reach the intended diagnosis.
- Accept the pharmacist's clinical advice neutrally. Do not argue, display clinical knowledge, or use technical terms that are not part of the script.
- Do not purchase a prescription medicine. If an over-the-counter product is offered, defer purchase using the standardized exit statement.
- Use the reporting-readiness probe only after the pharmacist has completed the initial clinical assessment and management. Clinical recognition and management scoring closes before the probe and cannot be changed by post-probe statements. Stop any reporting workflow before transmission.
- Exit immediately if personal safety is threatened, if the pharmacy becomes too crowded to continue confidentially, or if the pharmacist identifies the visit as research before completion. If the pharmacist attempts to activate ambulance/EMS services, state that family transport is already arranged; if an emergency call is initiated, disclose the simulation before dispatch. During debriefing, do not reveal future sites, route timing, or other SP identities, and request that scenario details not be circulated until fieldwork ends.

Standardized scenario scripts

Script component	Scenario A: Semaglutide	Scenario B: Empagliflozin	Scenario C: Apixaban
Simulated patient	Woman	Man	Man
Opening statement	'Since yesterday I have had very strong pain here in the upper stomach going toward my back, with nausea. I use this weekly injection and recently increased the dose. Can you give me something so I do not have to go to hospital?'	'I take this diabetes tablet. Since I became unwell and stopped eating properly, I feel extremely tired, nauseated, and have stomach pain. My breathing feels deep and fast. My glucose was 165. Can I take an antacid or rehydration solution?'	'I take apixaban for an irregular heartbeat. Since last night my stool has been black and I feel dizzy and weak. I also took ibuprofen for my back. Can you give me something for my stomach?'
Medicine if asked	Semaglutide 1.0 mg subcutaneously once weekly; increased from 0.5 mg 2 weeks ago	Empagliflozin 10 mg once daily; also basal insulin and metformin	Apixaban 5 mg twice daily for atrial fibrillation
Symptom detail if asked	Persistent 8/10 epigastric pain radiating to back; nausea; no diarrhea; one vomit; started 18 hours ago	Nausea, diffuse abdominal pain, marked fatigue, thirst, and deep breathing for 12 hours; no chest pain	Two black tarry stools; light-headed on standing; no bright-red blood; symptoms started overnight
Relevant history	No prior pancreatitis; no known gallstones; rare alcohol use; no pregnancy	Reduced food and fluid intake for 2 days during viral symptoms; missed one insulin dose; no ketone measurement	Ibuprofen 400 mg three times over 2 days; no other antiplatelet; last apixaban dose this morning
Requested product	Painkiller or antiemetic	Antacid or oral rehydration solution	Stomach medicine
Standard resistance	'Do I really need to go now? Could I wait until tomorrow?'	'But my sugar is not very high—could it still be serious?'	'Can I just stop the ibuprofen and wait?'

Script component	Scenario A: Semaglutide	Scenario B: Empagliflozin	Scenario C: Apixaban
Reporting-readiness probe	'Could this be a reaction to my medicine, and is it something you can help report?'	Same standardized probe	Same standardized probe
Exit statement	'I will call my family now and confirm all the medicine details before anything is submitted.'	Same standardized exit	Same standardized exit

The scripts were delivered in standardized Jordanian Arabic after linguistic review and local medicine-availability verification.

Table S1. Operational Definitions and Scoring Rubric

Primary outcome algorithm: safe triage = urgent ED/EMS referral AND explicit communication of urgency AND no unsafe delaying management. Adequate assessment = at least four of six domains: (1) medicine-and-dose, for which both suspected-product identity and dose/frequency had to be established; (2) treatment timing; (3) symptoms/course; (4) severity/red flags; (5) concomitant medicines; and (6) relevant history/risk factors. The ≥4/6 threshold is a pragmatic majority-of-domains standard endorsed by the expert panel; it rewards breadth without requiring an exhaustive history before emergency escalation. Allergy/prior ADR was collected and reported separately but was not part of the six-domain composite. Appropriate medicine-specific advice = withhold next semaglutide dose pending urgent review (GLP-1 receptor agonist scenario); temporarily withhold empagliflozin pending emergency assessment (SGLT2 inhibitor scenario); or avoid further NSAIDs and avoid independent alteration of apixaban outside urgent medical instruction (apixaban scenario). All clinical variables are scored only from the pre-probe segment. Pharmacovigilance scoring distinguishes spontaneous reporting discussion from responses after the standardized reporting-readiness probe. Where the checklist and free-text reconstruction disagree, the verbatim reconstruction takes precedence after blinded adjudication.

Variable	Code 1	Code 0
Medicine identity	Asked or confirmed generic/brand name or inspected package/photo	Asked only whether the patient takes 'medicines' without identifying the suspect product
Dose	Asked, confirmed, or verified the dose/strength and dosing frequency of the suspected medicine	Identified the suspected medicine but did not establish its dose/strength and frequency
Treatment timing	Asked start date, recent dose escalation, or temporal relation between last dose and symptoms	Asked how long the patient has diabetes/atrial fibrillation without treatment timing
Symptom onset/course	Asked when symptoms began and established duration and/or progression since onset	Asked only whether symptoms were present without timing, duration, or course
Red-flag assessment	Explored pain severity/radiation, breathing pattern, syncope/dizziness, vomiting, melena frequency, or inability to maintain intake as appropriate	Generic 'anything else?' with no red-flag follow-up
Concomitant medicines	Asked about other prescription and/or nonprescription medicines relevant to the encounter, including recent short-term use	No inquiry about other medicines or asked only about the suspected product
Relevant history/risk factors	Asked at least one scenario-relevant medical-history or risk-factor question (e.g., gallstones/alcohol, illness/reduced intake/insulin omission, bleeding risk)	No scenario-relevant medical-history or risk-factor inquiry
Allergy or prior ADR	Asked about drug allergy, previous adverse reaction, or prior similar medicine-related event	No inquiry about allergy, previous ADR, or prior similar medicine-related event
Medication-related recognition	Stated that the medicine may be causing or contributing to symptoms	Named the disease only with no medicine link
Specific recognition	Named pancreatitis; euglycemic/SGLT2-associated ketoacidosis; or GI/anticoagulant-related bleeding	Used only nonspecific terms such as 'side effect' or 'stomach problem'

Variable	Code 1	Code 0
Urgent referral	Directed the patient to an emergency department (ED) or emergency medical service (EMS), including arranging immediate transfer or emergency assessment through ED/EMS	Advised routine physician appointment, non-emergency assessment, laboratory testing later, or review only if symptoms worsened
Explicit urgency	Used 'now,' 'immediately,' 'today,' 'emergency,' or an equivalent unambiguous Arabic phrase	Referral without a timeframe
Unsafe delaying management	Reassurance, wait-and-see advice, or symptom-directed product positioned as an alternative to urgent care	Comfort advice explicitly subordinate to immediate emergency referral
Spontaneous reportability	Raised ADR reporting before the standardized probe	Discussed documentation only after the probe
Legitimate reporting route	Named JFDA electronic reporting or another legitimate channel that forwards a case to the national system	Told the patient only to inform the prescriber
Reporting assistance initiated	Opened form, supplied direct official link/QR, contacted PV service, or started minimum-data collection with stated intent to report	Agreed that reporting is useful but took no action
Minimum case elements	Elicited identifiable patient, identifiable reporter, suspected product, and suspected reaction	Any subset of three or fewer elements
Appropriate medicine-specific advice	GLP-1 RA: withhold next semaglutide dose pending urgent review; SGLT2 inhibitor: temporarily withhold empagliflozin pending emergency assessment; apixaban: avoid further NSAIDs and avoid independent apixaban changes outside urgent medical instruction	Symptom-directed medicine advice without the scenario-specific safety instruction
Prompted reporting readiness	After the standardized probe, acknowledged reportability or undertook a defined reporting action; analyzed only among encounters without spontaneous reporting discussion	Post-probe behavior is not counted as spontaneous reporting and cannot satisfy pre-probe clinical outcomes

Table S2. Simulated-Patient-Specific Descriptive Consistency Checks

Scenario	SP	Safe triage, n/N (%)	Medication-related recognition, n/N (%)	Specific syndrome/harm named, n/N (%)
GLP-1 RA / pancreatitis	S1	30/64 (46.9)	46/64 (71.9)	24/64 (37.5)
GLP-1 RA / pancreatitis	S2	24/64 (37.5)	44/64 (68.8)	17/64 (26.6)
SGLT2 inhibitor / euglycemic DKA	S3	17/64 (26.6)	39/64 (60.9)	15/64 (23.4)
SGLT2 inhibitor / euglycemic DKA	S4	11/64 (17.2)	31/64 (48.4)	8/64 (12.5)
Apixaban / GI bleeding	S5	38/64 (59.4)	61/64 (95.3)	46/64 (71.9)
Apixaban / GI bleeding	S6	40/64 (62.5)	59/64 (92.2)	42/64 (65.6)

SPs were structurally nested within scenarios; these results are descriptive consistency checks and are not intended to estimate an SP-independent scenario effect. Patient sex was also scenario-linked (woman in the semaglutide scenario; men in the empagliflozin and apixaban scenarios). With only two SPs per scenario and no SP crossing between scenarios, a cross-classified random-effects model cannot separately identify SP, patient-sex, and scenario effects; an SP-level intraclass correlation coefficient would therefore not have an interpretable independent scenario-adjusted meaning in this design.

Table S3. Appropriate Scenario-Specific Medicine Advice (Descriptive Process Measure)

Scenario	Operational advice criterion	Appropriate advice, n/N (%)
GLP-1 RA / pancreatitis	Withhold next semaglutide dose pending urgent clinical review	67/128 (52.3)
SGLT2 inhibitor / euglycemic DKA	Temporarily withhold empagliflozin pending emergency assessment	54/128 (42.2)

Scenario	Operational advice criterion	Appropriate advice, n/N (%)
Apixaban / GI bleeding	Avoid further NSAIDs and avoid independent apixaban changes outside urgent medical instruction	82/128 (64.1)
Overall	Scenario-specific criterion applied within each assigned scenario	203/384 (52.9)

Because the required medication action differed across the three clinical scenarios, these results are reported descriptively and are not included in the cross-scenario FDR family.

Table S4. Reporting Assistance Initiated by End of Encounter According to Clinical Performance

Clinical subgroup	Reporting assistance initiated, n/N (%)	Interpretive note
Medication-related cause recognized	38/280 (13.6)	Pre-probe recognition present
Medication-related cause not recognized	8/104 (7.7)	Pre-probe recognition absent
Safe triage achieved	26/160 (16.3)	All three primary components satisfied
Safe triage not achieved	20/224 (8.9)	At least one primary component absent

These cross-tabulations are descriptive and are not adjusted for scenario or other covariates.

Table S5. Organization-Clustered Sensitivity Analysis for the Safe-Triage Model

Predictor	aPR	Primary robust 95% CI	Organization-clustered 95% CI	Clustered p value
Empagliflozin vs semaglutide	0.51	0.35–0.74	0.35–0.74	<.001
Apixaban vs semaglutide	1.54	1.21–1.96	1.22–1.94	<.001
Chain vs independent ownership	1.03	0.81–1.30	0.81–1.30	.822
High vs low/moderate workload	0.53	0.39–0.72	0.40–0.71	<.001
Visible vs no consultation area	1.32	1.05–1.67	1.03–1.69	.026
Recent PV training: yes vs no	1.33	1.01–1.74	1.01–1.74	.040
Female vs male pharmacist	1.04	0.83–1.29	0.83–1.30	.762
Zarqa vs Amman	1.08	0.84–1.39	0.83–1.42	.560
Al-Balqa vs Amman	0.95	0.68–1.34	0.68–1.33	.783
Evening vs daytime	0.97	0.77–1.22	0.76–1.24	.796

Point estimates are identical because the model specification and fitted coefficients are unchanged; only the covariance estimator differs. Organization-clustered sandwich covariance used 298 clusters: 287 independent pharmacies as singleton clusters plus 11 chain organizations containing 97 sampled branches. aPR, adjusted prevalence ratio; CI, confidence interval; PV, pharmacovigilance. The 298-cluster sensitivity analysis primarily evaluates residual organization-level dependence: 287 clusters are independent singleton pharmacies and only 11 clusters contain multiple sampled branches. Results are therefore interpreted as a covariance sensitivity analysis rather than estimation of a chain-level random effect.

Table S6. Extreme-Case Sensitivity Bounds for Post-Encounter Consent Refusals

Extreme-case assumption	Safe-triage numerator	Denominator	Safe-triage prevalence
All 19 consent decliners assumed not to have achieved safe triage	160	403	39.7%
All 19 consent decliners assumed to have achieved safe triage	179	403	44.4%

These deterministic bounds use the 384 retained encounters plus the 19 completed encounters excluded solely because post-encounter consent for data retention was declined (n = 403 otherwise retainable completed encounters). They are intended as an assumption-free extreme-case sensitivity analysis and do not reconstruct or impute the destroyed records.
